# Supplementary material for: A large fraction of neocortical myelin ensheathes axons of local inhibitory neurons
Source: eLife. 2016 Jul 6;5:e15784. doi: 10.7554/eLife.15784 (PMC4972537; doi:10.7554/eLife.15784)
Supplement: Supplementary file 1. — Pearson’s correlation coefficients from 4 different control experiments are shown. The comparison between adjacent sections tests the consistency of staining, as the distribution of targets is very similar on two adjacent ultrathin sections (70 nm thickness). This correlation is influenced by antibody characteristics, but also the size of targets, with smaller targets displaying larger spatial variability from section to section. This could explain the lower R for NF-H which labels axons, as some axons can be very thin (<100 nm). The comparison with an antibody against an overlapping antigen is a test for the specificity of staining. The following comparisons were done: MBP/PLP, GABA/GAD2, PV/GABA, NFHch/NFHr, αTub/ac αTub. The lower coefficients for GABA/GAD2 and PV/GABA reflect the fact that GAD2 and PV are present in only a subset of GABA containing structures. Another test for specificity is the comparison with an antibody against a spatially exclusive antigen. Values of R around 0 are expected in this case. MBP and PLP (present in the myelin sheath) were each compared with GABA (inside inhibitory neurons); GABA and PV (inhibitory neurons) were compared with VGluT1 (excitatory neurons); NFH, αTub and ac αTub (all predominantly neuronal) with glutamine synthetase (glial). And finally, all antibodies were compared with DAPI to control for background nuclear staining. DOI: http://dx.doi.org/10.7554/eLife.15784.025 [file elife-15784-supp1.docx]

**Supplementary File 1: Antibody controls**

|  | **MPB** | **PLP** | **GABA** | **PV** | **NFH** | **αTub** | **ac αTub** |
| --- | --- | --- | --- | --- | --- | --- | --- |
| **Adjacent section** | 0.91 | 0.77 | 0.92 | 0.67 | 0.48 | 0.77 | 0.74 |
| **Overlapping antigen** | 0.92 | 0.92 | 0.52 | 0.34 | 0.71 | 0.75 | 0.75 |
| **Exclusive antigen** | 0.09 | 0.07 | -0.01 | -0.02 | 0.01 | -0.12 | -0.12 |
| **Nuclear** | -0.07 | -0.05 | -0.06 | -0.04 | -0.01 | -0.26 | -0.29 |

| -0.4 | -0.2 | 0 | 0.2 | 0.4 | 0.6 | 0.8 | 1 |
| --- | --- | --- | --- | --- | --- | --- | --- |

**Antibody controls.** Pearson’s correlation coefficients from 4 different control experiments are shown. *The comparison between adjacent sections* tests the consistency of staining, as the distribution of targets is very similar on two adjacent ultrathin sections (70 nm thickness). This correlation is influenced by antibody characteristics, but also the size of targets, with smaller targets displaying larger spatial variability from section to section. This could explain the lower R for NF-H which labels axons, as some axons can be very thin (<100 nm). The comparison with an antibody against an *overlapping antigen* is a test for the specificity of staining. The following comparisons were done: MBP/PLP, GABA/GAD2, PV/GABA, NFHch/NFHr , αTub/ac αTub. The lower coefficients for GABA/GAD2 and PV/GABA reflect the fact that GAD2 and PV are present in only a subset of GABA containing structures. Another test for specificity is the comparison with an antibody against a spatially *exclusive antigen*. Values of R around 0 are expected in this case. MBP and PLP (present in the myelin sheath) were each compared with GABA (inside inhibitory neurons); GABA and PV (inhibitory neurons) were compared with VGluT1 (excitatory neurons); NFH, aTub and ac a Tub (all predominantly neuronal) with glutamine synthetase (glial). And finally, all antibodies were compared with DAPI to control for background *nuclear* staining.
